# High temperature requirement A1 in cancer: biomarker and therapeutic target

**DOI:** 10.1186/s12935-021-02203-4

**Published:** 2021-09-25

**Authors:** Mingming Chen, Shilei Yang, Yu Wu, Zirui Zhao, Xiaohan Zhai, Deshi Dong

**Affiliations:** 1 Department of Pharmacy, The First Affiliated Hospital of Dalian Medical University, 222, Zhongshan Road, Xigang District, 116011 Dalian, China; 2 Department of Clinical Pharmacology, College of Pharmacy, Dalian Medical University, Dalian, China

**Keywords:** High temperature requirement A1, Cancer, Biomarker, Therapeutic target

## Abstract

As the life expectancy of the population increases worldwide, cancer is becoming a substantial public health problem. Considering its recurrence and mortality rates, most cancer cases are difficult to cure. In recent decades, a large number of studies have been carried out on different cancer types; unfortunately, tumor incidence and mortality have not been effectively improved. At present, early diagnostic biomarkers and accurate therapeutic strategies for cancer are lacking. High temperature requirement A1 (HtrA1) is a trypsin-fold serine protease that is also a chymotrypsin-like protease family member originally discovered in bacteria and later discovered in mammalian systems. HtrA1 gene expression is decreased in diverse cancers, and it may play a role as a tumor suppressor for promoting the death of tumor cells. This work aimed to examine the role of HtrA1 as a cell type-specific diagnostic biomarker or as an internal and external regulatory factor of diverse cancers. The findings of this study will facilitate the development of HtrA1 as a therapeutic target.

## Background

Cancer is among the main health issues worldwide and is also the main cause of mortality in China [1, 2]. In 2018, 4,285,033 cancer patients were diagnosed, and 2,865,174 cancer-related deaths were reported by the Global Cancer Observatory [3]. Cancer represents a malignant disorder characterized by aggressive and out-of-control cell growth resulting from increased expression of tumor-enhancing genes and decreased expression of tumor suppressor genes [4]. Limited by the atypical early symptoms and the lack of early sensitive and specific diagnostic markers, most cancer cases are diagnosed at advanced stages, and the best opportunity for surgery is missed [5, 6]. Despite the continuous advancement of radiotherapy and chemotherapy, the incidence and mortality of malignant tumors have not been significantly reduced. Thus, studies of early diagnostic markers and novel targets for treatment development are urgently needed.

High-temperature requirement A (HtrA) family members are homo-oligomeric serine proteases with a high degree of conservation that participate in diverse mammalian cell processes, such as proliferation [7], mitochondrial homeostasis [8], apoptosis [9], and protein quality control [10]. The dual effects of bacterial HtrA proteases as high-temperature proteases or low/normal-temperature chaperone proteins have been extensively studied [11]. HtrA family members have vital roles in the activation of cell stress responses, and these proteins have also been suggested to enhance the proteolytic activity necessary for degrading periplasmic misfolded proteins [10, 12, 13]. Additionally, certain mammalian HtrA proteins have been identified as possible regulators of chemotherapy-mediated cytotoxicity and programmed cell death [9]. On the other hand, HtrA proteases have been related to carcinogenesis, and their expression is decreased in ovarian cancer (OC) [14], thyroid cancer (TC) [9], endometrial carcinoma (EC) [15], breast cancer (BC) [16], hepatocellular carcinoma (HCC) [17], and colorectal cancer (CRC) [18].

There are 4 members of the human HtrA family, namely, HtrA1–4. In human HtrA1, there are 4 well-recognized domains, including one PDZ domain, one IGFBP domain, one protease domain and one Kazal domain. HtrA1 is an extensively studied secretory protein that is distributed in cells and related to microtubules [19]. HtrA1 promotes cartilage degradation by degrading extracellular matrix in the pathology of arthritis [20]. A significant increase in HtrA1 expression during late pregnancy, especially in syncytiotrophoblasts, leads to ischemia and hypoxia of the placenta, which has become one of the etiologies of hypertension in pregnancy [21]. Polymorphisms in the promoter region of HTRA1 are strongly associated with age-related macular degeneration (AMD) [22]. Therefore, regulation of the expression and protease activity of HtrA1 is an opportunity for preventing life-threatening illnesses, such as skeletal disorders, AMD, and neuropathological disease. Existing studies suggest that HtrA1 protects against diverse malignant tumors because of its antitumor activity [23]. Promoter methylation-mediated HtrA1 downregulation induces diverse phenotypes that may serve as cancer cell hallmarks; therefore, HtrA1 may be used as a biomarker for malignant transformation or tumor development [23]. Here, we review the actions of HtrA1 in the pathogenesis of cancers, which may contribute to the development of therapeutic agents targeting HtrA1 in tumorigenesis.

### Gynecological cancers

#### Endometrial carcinoma

EC, a frequently occurring malignancy in the genital tract of women, usually affects postmenopausal women [24]. Notably, endometrioid carcinoma is the most frequently observed EC type (approximately 80% of EC cases) and is related to endometrial hyperplasia [24]. At present, 2 distinct clinicopathological subtypes of EC have been discovered, namely, the estrogen- and nonestrogen-associated subtypes (referring to type I and type II, endometrioid and nonendometrioid, respectively) [25]. Although EC has been studied for years, its molecular basis remains incompletely understood, and there is currently no specific test to screen EC. There is no useful assay to diagnose EC or evaluate the treatment response of EC. Therefore, more genetic and biochemical studies should be conducted to reveal the biology of EC and to predict its prognostic outcome.

A loss of heterozygosity (LOH) at DMBT1 occurs in 50% of EC cell lines [26]. DMBT1 is a potential tumor suppressor that is located on 10q26.13 and that is characterized by intrasolar homozygous deletions and rare mutations [27]. The human HTRA1 gene is located on chromosome 10q25.3-q26.2, which is very close to DMBT1, suggesting HtrA1 as a presumed tumor suppressor [28]. The expression of the HTRA1 and HTRA3 genes is detected within mouse embryonic organs in a complementary pattern [29]. The highest expression of HTRA1 is observed in the placenta, but its expression is relatively low compared to that of HTRA3 in other organs such as fetal heart, heart, and ovaries [30]. Consistently, HTRA1 and HTRA3 are differentially expressed within OC cells. The gene abundance of HTRA1 and HTRA3 is decreased to varying degrees with increasing EC grade. The difference is that HTRA3 mRNA levels gradually decline as tumor grade increases, while HTRA1 mRNA levels immediately decrease with the increase in early G1EC, then remain at a lower level, and then dynamic change with the increase in tumor grade. Presumably, a low level of HTRA1 mRNA predicts early G1EC, but a low level of HTRA3 mRNA is a more reliable predictor of late G3EC. In light of the differences in the HTRA1 and HTRA3 sequences, identifying subtype-specific substrates will shed more light on the real functions of HTRA1 and HTRA3 in EC.

#### Ovarian cancer

OC ranks 7th among female cancers in terms of its morbidity. OC is a fatal gynecological malignancy, and over 75% of cases are diagnosed at advanced stages because of a lack of symptoms; its 5-year survival postdiagnosis is 46% [5]. In the USA, OC is a fatal gynecologic tumor, which affects approximately 22,000 patients and results in 16,000 deaths every year [31]. Over 80% of patients originally respond to chemotherapy and surgery, but over 75% of patients ultimately die due to disease relapse or chemoresistance [32]. Thus, it is of great importance to formulate new treatment options to overcome chemoresistance to improve patient survival.

HtrA1 expression was not detected or is detected at a low level in 59% of primary OCs relative to that in the ovarian surface epithelium. High loss-of-allele frequency is detected in microsatellite markers close to HTRA1 locus on 10q26 in the context of primary OC [14]. Moreover, HtrA1 expression is decreased in 5/7 OC cell lines, including OSE50, OV202, OV207, OVCAR5 and SKOV3. Consistent with the concept that HtrA1 is a tumor suppressor, HtrA1 expression downregulation in SKOV3 cells after antisense transfection enhanced anchored independent growth [14]. Although it is currently recognized that HTRA1 expression is reduced in different types of ovarian carcinoma, in high-grade serous OC, nuclear expression of cleaved HtrA1 is associated with good prognosis [33, 34].

**Fig. 1:**
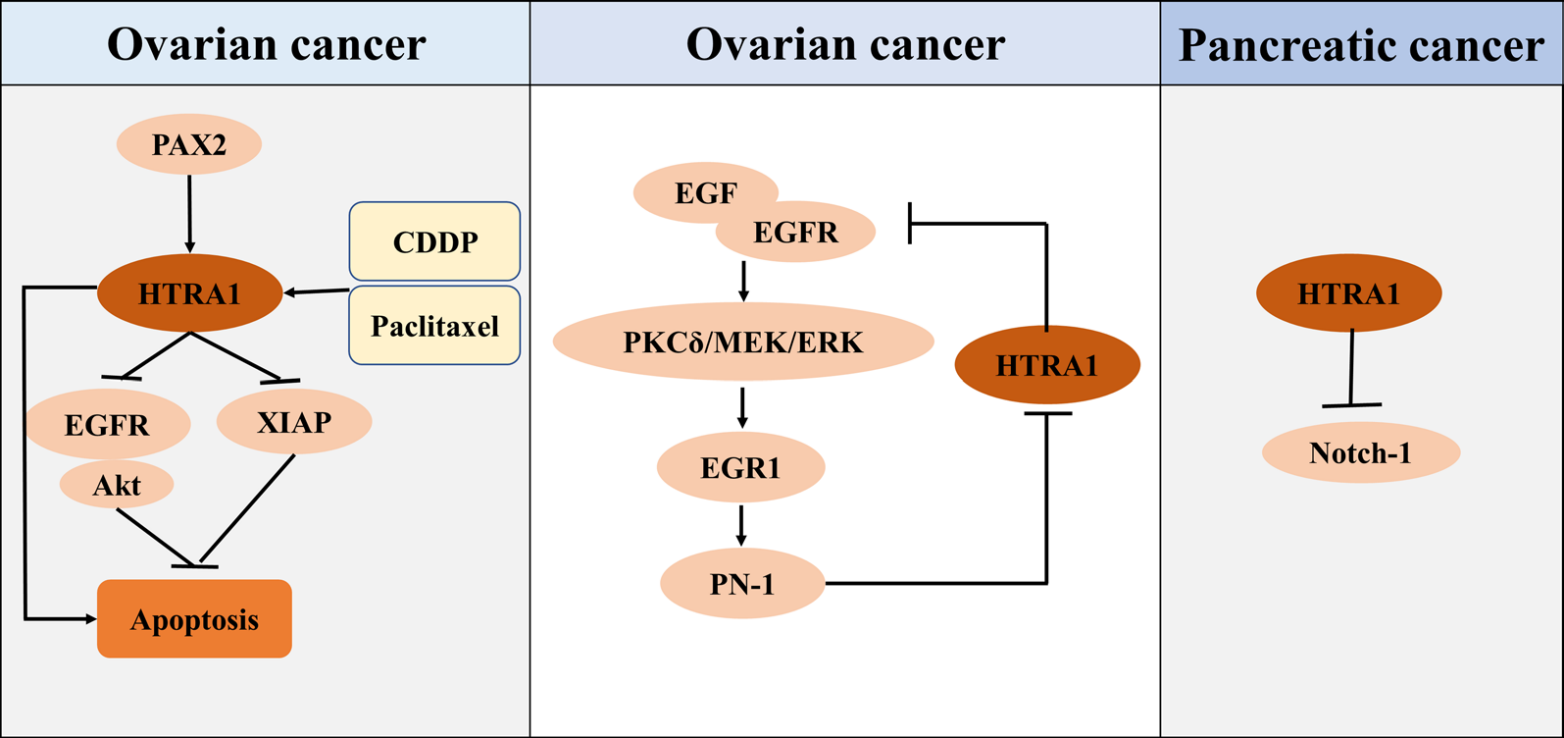
The potential mechanisms underlying the effects of HTRA1 on EOC, BC and PC. In EOC, HtrA1 mediates apoptosis by inhibiting the levels of nuclear EGFR and XIAP, X-conjugated inhibitor of apoptosis proteins. In BC, PN-1 expression is regulated through the EGF/EGFR/PKCδ/MEK/ERK/EGR1 signaling pathway to inhibit the effect of the HTRA1 protein on tumors. In PC, HTRA1 directly inhibits the Notch-1 signaling pathway to regulate the tumor microenvironment

Patients with OC mainly die from metastasis. Epithelial cells constitute the physiological barrier against the development of metastasis and undergo the process of cell death known as “anoikis” because they do not contact the extracellular matrix (ECM) [35]. Therefore, OC cells must acquire nest-loss apoptosis resistance for their survival within ascites prior to the formation of metastases. HtrA1 has been identified as a proapoptotic factor in OC. Overexpression of full-length HtrA1 can promote OC cell apoptosis [36]. A growing body of studies suggest the involvement of nuclear EGFR in some diverse cell processes that are important for cancer development, such as cell proliferation-related gene transcription, DNA repair and biosynthesis, and cell chemoresistance [37, 38]. In recent years, the upregulation of nuclear EGFR has been suggested to predict dismal prognostic outcomes in OC [39]. Nuclear colocalization of EGFR and HtrA1 indicates the role of nuclear HtrA1 in regulating nuclear EGFR, which may affect OC metastasis. Mechanistically, HtrA1 acts upstream of EGFR, which attenuates the activation of the EGFR/Akt pathway and ultimately promotes nest-loss apoptosis. p-EGFR expression markedly is increased in xenograft tumors with downregulated HtrA1 expression, further confirming this inhibition in vivo [37, 38]. High PAX2 expression is observed in low malignant potential and low-grade OC but not in normal ovarian tissues. In normal murine OSE cells (mOSE) transformed with K-RAS and c-MYC, PAX2 exhibits oncogenic properties by enhancing pERK1/2 and COX2 expression with loss of p53 expression; however, PAX2 reduces proliferation and metastasis in high-grade serous OC by increasing HtrA1 expression and decreasing COX2 expression [40]. X-linked inhibitor of apoptosis protein (XIAP), a member of the inhibitor of apoptosis proteins (IAPs) family, performs a vital function in regulating apoptosis. XIAP protects cells against death due to various cellular attacks through the direct suppression of caspase cascade initiation and execution [41]. XIAP levels are recognized to be a vital factor for cell survival in OC, and XIAP endows cells with resistance to cisplatin (CDDP)-induced apoptosis [42–45]. HtrA1-mediated apoptosis is dependent on serine protease activity, indicating that there is an unknown substrate related to chemoresistance [12]. A consistent cleavage site for HtrA1 has been identified through mixture-oriented peptide library screening, where XIAP is matched as the possible HtrA1 substrate [46]. Purified wild-type (WT) HtrA1, rather than mutant (mut) HtrA1, can degrade recombinant XIAP in vitro. Consistent with data in vitro, XIAP and HtrA1 were found to form a complex in vivo, as revealed by coimmunoprecipitation (Co-IP) assays. Ectopic HtrA1 expression reduced XIAP expression in OV202 and OV167 cells, whereas HtrA1 silencing led to XIAP expression upregulation in SKOV3 cells [47]. HtrA1 expression was reported to increase with CDDP- and paclitaxel-induced cytotoxicity. Both CDDP and paclitaxel enhance the overexpression of HtrA1, which in turn leads to limited self-proteolysis and activation of HtrA1, inducing cell death in a serine protease-dependent manner. Patients with higher HtrA1 expression levels showed a higher tumor response rate to chemotherapy than patients with lower expression levels [12] (Fig. 1).

#### Breast cancer

BC is the female cancer with the highest morbidity rate worldwide, and at present, there is no radical treatment for metastasis [48]. BC is a frequently occurring cancer in females and a major reason leading to mortality; approximately 508,000 women die annually from BC [49]. In developed countries, BC has a generally favorable prognosis, but the situation in developing countries is not favorable, and locally advanced BC is reported to be associated with a high morbidity rate [50]. According to histological and molecular findings, BC is divided into 3 types, namely, hormone receptor-positive EC (progesterone receptor (PR+) and estrogen receptor (ER+)), human epidermal receptor 2-positive EC (HER2+), or triple-negative breast cancer (TNBC, ER-, PR-, HER2-) [51]. Treatment of BC cancers must be selected according to the molecular features of BC. Additionally, TNBC can be classified into 6 types: basal-like 1 (BL-1), basal-like 2 (BL-2), mesenchymal (M), immunomodulatory (IM), luminal androgen receptor (LAR) and mesenchymal stem cell-like (MSL) [52]. Developing new treatments is of great necessity for improving patient survival.

Recently, downregulated HTRA1 mRNA expression was detected in BC cases, showing aggressive clinical characteristics [53]. Based on Cox proportional hazard models, the high expression of HTRA1 is associated with favorable overall survival (OS) and disease-free survival (DFS), particularly in node-positive patients [16]. HTRA1 is significantly expressed within the normal ductal glands in the breast, but HTRA1 expression is significantly downregulated or disappears within the tumor tissues of those with invasive BC or ductal carcinoma in situ (DCIS) [54]. Furthermore, HTRA1 was discovered, with an additional 2 genes, MTSS1 and CLPTM1, to be an indicator of doxorubicin-sensitive disease among nonresponsive BC cases in 95% of samples [55]. A retrospective study of 333 nonmetastatic patients with locally advanced BC who underwent neoadjuvant chemotherapy (NACT) showed that high HTRA1 expression may indicate a lack of response to NACT and a predictor of increased risk of cancer recurrence and death [56].

In BC, Wang et al. recently showed that HTRA1 deficiency is actually accompanied by stromal characteristic acquisition [54]. It is hypothesized that HtrA1 plays an important role in modulating the stability and dynamics of microtubule assembly due to intracellular HtrA1 colocation and binding to microtubules via the PDZ domain. HtrA1 upregulation weakens cell motility, while loss of HtrA1 expression promotes cell motility [57]. Epithelial-mesenchymal transformation (EMT) is characterized by elevated aggressiveness and motility. In samples from patients who progressed to metastasis after NACT, the potential 6-EMT gene signature, including LUM, SFRP4, COL6A3, MMP2, CXCL12, and HTRA1, was consistently expressed at higher levels [58]. The reduction of HtrA1 expression promotes EMT and contributes to the acquisition of a mesenchymal-like phenotype, such as promoted proliferation rate, invasion and migration ability and higher expressions of mesenchymal biomarkers. Additionally, decreased HtrA1 expression activated DNA damage response (DDR) and ataxia telangiectasis mutated (ATM), while upregulation of HtrA1 expression prevents DDR and ATM [54]. In vitro, knockdowning of HTRA1 by siRNA suppressed breast epithelial cells migration and invasion [57]. Protease nexin-1 (PN-1) increased the migration, invasion and stemness of BC cells via the EGF/EGFR/PKCδ/MEK/ERK/EGR1 axis. In breast tumorigenesis, EGF, which is increased in the tumor microenvironment, upregulates the expression of PN-1 through binding to EGFR and followed by the activation of downstream kinases, such as ERK, PKCδ, MEK, and its transcription factor EGR1. PN-1 will block the function of HtrA1, that is, disrupt EGF cleavage, resulting in further activation of the EGF signal as a feedback signal to upregulate PN-1 expression [48] (Fig. 1).

#### Cervical cancer

Cervical cancer is one of the most common malignant tumors in women, and its morbidity and mortality rates rank first among those of female reproductive system malignant tumors. Human papilloma virus (HPV) is a DNA virus, and persistent HPV infection contributes to 99% of the malignant transformation of cervical epithelial cells [59]. Stuqui et al. showed that HTRA1 overexpression does not affect apoptosis in either HPV-negative (C33) or HPV16-positive (CasKi) cervical cells but interferes with cell proliferation. More CasKi cells with HTRA1 overexpression are arrested in the S phase, while more HTRA1-transfected C33 cells are arrested in the G0/G1 phase [60].

### Gastrointestinal cancer

#### Esophageal cancer

Esophageal cancer is a common malignant tumor in some countries and regions of the world. China is one of the countries with a high incidence of esophageal cancer and a high mortality rate of esophageal cancer worldwide. Compared with that in adjacent tissue, HTRA1 mRNA and protein expression in esophageal carcinoma is significantly decreased, especially in highly undifferentiated esophageal tumor tissue. In addition, HtrA1 expression has a significant negative correlation with pathological stages and lymph node metastasis but a significant positive correlation with the survival rate of patients [61, 62]. Elevated HtrA1 expression inhibits the phosphorylation of IκBα and p65, which is coupled to decreases in Ki-67, Bcl-2, Bcl-xL, cyclin D1, and MMP-9 protein expression [62].

#### Gastric cancer

In many parts of the world, the total numbers of gastric cancer patients and related deaths are increasing each year. For East Asia, China is a country with high gastric cancer occurrence rates, and its death rate and DALY remain high. Compared with the general issue, gastric cancer (GC) tissue expresses lower HtrA1. It is predicted that when HtrA1 is detected before chemotherapy, platinum-based chemotherapy for gastric cancer may be needed. The median overall survival (OS) of patients who suffer from high or medium HtrA1 expression is 17 months, while that of patients who suffer from low HtrA1 expression is 9.5 months [63]. Mechanistically, HtrA1 inhibits gastric carcinoma cells, and weakened the effect of cell in proliferating, invading, and migrating [64].

Carcinoma-associated fibroblasts (CAFs), a vital part of tumor stroma, supports epithelial cells physiologically and play a pivotal role in the functionality regulation of tumor invasion, metastasis and poor prognosis, promoting and delaying tumor development in an environment-dependent manner [65, 66]. Tumor cells and fibroblasts can secret a variety of cytokines and growth factors, including il-6, bFGF/FGF2, TGF-β1 and HGF, which triggers the transdifferentiation of nontransgenic normal fibroblasts (NFSs) to CAFs [67, 68]. After transdifferentiation, CAFs are associated with high-level protein biomarkers, particularly α-smooth muscle actin (α-SMA) [69, 70]. As HtrA1 expression in gastric carcinoma cell line is up-graduated, α-SMA expression in normal fibroblasts will be increased. According to the studies, overexpression of HtrA1 can significantly increase bFGF/FGF2 secretion by gastric cancer cells by activating NF-кB signal [71]. Gene sequence analysis showed that the HTRA1 promoter supermethylated region includes a variety of transcription factor binding sites, such as c-Myc, AP-1, and E2F. Therefore, epigenetic silencing of the HTRA1 gene enables the reactivation of HTRA1 gene expression in gastric carcinoma cells [72].

#### Pancreatic cancer

Pancreatic cancer (PC) results in 331,000 deaths annually, and it ranks 7th among the causes of cancer-associated mortality in the general population [73]. Many efforts have been made in recent years, but the 5-year survival rate is as low as 5% [74]. Typically, tobacco smoking is identified as a cause of PC, which may account for certain gender differences as well as international variations [75]. PC has a high mortality rate since there are no effective early diagnostic markers or efficient treatments for advanced cancers [76]. Therefore, it is important to further identify new therapeutic targets to successfully treat PC.

HTRA1 mRNA is expressed at low levels in PC tissues compared with matched noncarcinoma tissues. Similarly, HtrA1 expression is decreased in PC cells relative to noncarcinoma pancreatic epithelial cells [77]. Notch signaling performs an important function in cell proliferation, differentiation, migration and apoptosis. Changes in Notch signaling are suggested to be related to carcinogenesis. An increasing number of studies have shown aberrant Notch-1 expression within certain malignant tumors, particularly PC [78–80]. Recent research found that the antiproliferative effect of HTRA1 in PC is dependent on Notch-1. The upregulation of HtrA1 expression inhibits Notch-1 expression in PC cells, which can be reversed by HtrA1-specific siRNA knockdown. Notch-1 overexpression further reverses the inhibitory effect of HtrA1 on tumor cell growth (Fig. 1) [77].

#### Colorectal cancer

In China, the morbidity of colorectal cancer (CRC) ranks only second to that of lung cancer and stomach cancer, all of which are deemed as the most common cancers. A low HtrA1 expression in cancer tissues is related to the poorer survival of CRC patients [81]. In an analysis of tissue specimens from Caucasian Italian subjects, HtrA1 expression was negatively correlated with ulcerative colitis duration and functioned as a biomarker to identify patients with ulcerative colitis of > 10 years duration (UCL) who were at high risk of developing CRC [18]. Additionally, the methylation status of the HTRA1 catalyst is a biomarker concerning tumor cells or cells to be transformed. Epigenetic silencing of HTRA1 by the epigenetic adaptor protein MBD2 accelerates late the growth of cells, amplifies the centrosome, and makes colon carcinoma cells polyploid [23].

Classic Wnt signaling pathways (also called Wnt/β-catenin signaling pathway) consist of specific Wnt ligands and their specific receptor-mediated interactions with β-catenin. Changes in Wnt structure from typical to abnormal proliferates the cells irregularly and aggravates various human cancers, especially human colorectal cancer [82, 83]. Studies have shown that HtrA1 is a new inhibiting factor of typical Wnt pathway. Inhibition of Wnt/β-catenin signal transduction by HtrA1 has an impact on the expression of multiple Wnt target genes through paracrine and autocrine pathways. In addition, HtrA1 generates a complex together with β-catenin, resulting in decreased cell proliferation [84].

HtrA1 expression is increased inSW480, a CDDP-cultured human colonic cancer cell line. The expression level of HtrA1 was decreased by continuous exposure of SW480 cells to CDDP. Instead, HtrA1’s ectopic expression in SW480/CDDP cells lowered XIAP expression and deactivated the PI3K/Akt signal pathway to eliminate the resistance of CDDP. XIAP interference inhibited CDDP resistance in SW480/CDDP cells [85], directly bound to caspase-3 and caspase-7 via the second baculoviral IAP repeat (BIR) domain (BIR2) region, and inhibited caspase-9 via the third BIR domain (BIR3) region [86].

#### Liver cancer

Liver cancer is the fifth most common cancer in the world. In China, the death rate of liver cancer now ranks second among the death rates of malignant tumors. HtrA1 expression in tumor tissues is downregulated than that in adjacent liver tissues. Patients expressing higher HtrA1 levels show a higher survival rate compared with those with lower HtrA1 levels [87]. Moreover, inverse relationships have been reported between HtrA1 expression and the differentiation of HCC and lymph node metastasis due to downregulation of HtrA1 expression and significantly increased the migration of cells. The expression of Vimentin and E-cadherin were decreased [17]. High HtrA1 expression targets XIAP to reverse the multidrug resistance of hepatoma cells; therefore, HtrA1 may be an effective target in HCC therapy [88].

### Lung cancer

Lung cancer is a disease that seriously endangers human health. In terms of incidence and mortality, both the lung cancer in China ranks first worldwide. According to immunohistochemistry of human lung cancer specimens, the expression of HtrA1 is significantly downregulated in metastasis of primary tumors and lymph node, which means that it is possibly associated with the progression of lung cancer [89]. HTRA1 is a CDDP resistance-related gene. In human nonsmall cell lung cancer (NSCLC), the targeting of HDAC/RXR/HtrA1 signaling axis may increase HtrA1 expression and overcome CDDP resistance [90]. PI3K/Akt signaling pathway is related to chemotherapy resistance in tumors. There have been a lot of studies on the structural activation of the PI3K/Akt signaling pathway regarding different cancers [91]. Inhibition of HTRA1 expression induces tumor stem cell characteristics in samples and CDDP resistance through the PI3K/Akt signal pathway [92].

### Lymphoma

Splenic marginal zone lymphoma (SMZL) is a rare type of lymphoma [93]. Inhibition of DNA promoter methylation is associated with the pathogenesis of B-cell lymphoma and may affect the prognosis of patients [94–96]. Research has integrated the whole genome DNA promoter methylation spectrum and gene expression spectrum, as well as clinical and biological variables. The high promoter methylation (high-M) group had a lower total survival rate than the low promoter methylation (low-M) group. In the high-M group, many tumor suppressive genes were methylated and suppressed, but only three genes (KLF4, CACNB2 and HTRA1) seemed to be able to identify high-risk cases, while abnormal DNA methylation seemed to play a role in influencing important biological pathways [97].

## Conclusions

HtrA1, which is a tumor suppressor, mediates the proliferation, migration, and invasion of cancer cells via a series of signals in the tumor progression and the microenvironment. To fully illustrate the mechanisms through which HtrA1 involved in cancers, it is necessary to further investigate the underling mechanism of HtrA1 as a cell type-dependent internal and external regulator. In particular, illustrating the mechanism of HtrA1 regulation, expression and protease activity will provide us a novel strategy in targeted therapeutics. The identification of HtrA1 substrates is also critical to gain insight into how to target this novel pathway efficiently, and it will be vital in clinic to determine the underlying mechanism that HtrA1 expression is regulated in chemotherapy. Generally, this review will arouse the interests of researchers in this novel pathway and jointly develop a novel and efficient approach for cancer cells targeted therapeutics.

## Data Availability

Not applicable.
